# Flow of Time: Perceiving the passage of time: neural possibilities

**DOI:** 10.1111/nyas.12545

**Published:** 2014-09-25

**Authors:** Timothy Muller, Anna C Nobre

**Affiliations:** Department of Experimental Psychology, Oxford Centre for Human Brain Activity, University of OxfordOxford, United Kingdom

**Keywords:** brain, time, causality, temporal illusion

## Abstract

Although the study of time has been central to physics and philosophy for millennia, questions of how time is represented in the brain and how this representation is related to time perception have only recently started to be addressed. Emerging evidence subtly yet profoundly challenges our intuitive notions of time over short scales, offering insight into the nature of the brain's representation of time. Numerous different models, specified at the neural level, of how the brain may keep track of time have been proposed. These models differ in various ways, such as whether time is represented by a centralized or distributed neural system, or whether there are neural systems dedicated to the problem of timing. This paper reviews the insight offered by behavioral experiments and how these experiments refute and guide some of the various models of the brain's representation of time.

## Introduction

Whether time exists or not, it cannot be denied that we subjectively perceive a passage of time. This raises the question of how the brain generates the subjective sense of time. We have no “time receptors,” as we do for other features of perception, such as shape, color, and pitch—so how does the brain keep track of time? This issue is of great importance, since timing is essential to all aspects of behavior: from structuring actions to planning the events in our lives. It is perhaps, therefore, surprising that the neural underpinnings of timing have remained relatively unexplored, for example, in comparison to the investigation of spatial cognition. As neuroscience begins to grapple with the fourth dimension, different possible mechanisms for neural timekeeping are beginning to be proposed and investigated. These models, along with psychophysical experiments that inform them, will be discussed in this review.

### Conceptions of time in physics and philosophy

Time is arguably one of most difficult entities to define. Indeed, even labelling it as an “entity” is loaded, since it implies it has an existence per se. Its elusive nature can be gleaned from a brief sketch of some notions of time offered by philosophy and physics.

Perhaps most appealing to our intuitions is the notion held by Newton stating that time exists: time is “absolute, true, and mathematical, in and of itself and of its own nature, without reference to anything external, flows uniformly and by another name is called duration.”^1^ This view states that time flows in a predictable manner, as we believe we perceive it.

A contrasting view with origins dating back to the sophists, then articulated by Leibniz, states that time has no independent existence, but rather it is “merely relative… an order of successions.”^2^ This view of time, as the structured relation among entities, rather than an entity itself, was taken further by Kant, who proposed that time, like space, had no external existence at all, but was instead one of the core organizing principles through which we organize sense data into a coherent perception.^3^ Yet stranger notions of time are captured in Einstein's theories of special and general relatively, positing that time is deeply meshed with space into a dynamic and curving space-time fabric.^4^

### Concepts of time in the brain

A few key concepts frame the literature on how time is represented in the brain. The first issue is whether time is represented in a centralized system^5^–^7^ or is distributed throughout the brain. The second issue is whether there are specialized neural systems exclusively dedicated to the problem of timing.^8^,^9^ Such a system could be defined as a dedicated “clock” within the brain. Dedicated timing mechanisms are contrasted with the alternative view that the circuits responsible for timing primarily process other information, such as other stimulus attributes^10^ or motor programs,^11^ but also intrinsically represent time. In this case, neural processes occur in time, without necessarily coding time per se. As is discussed in more detail below, the time dependency of these processes can give the illusion that the process is the coding of time per se. Therefore, apparently dedicated timing mechanisms may in fact be incidental to the processing of other attributes. This distinction between dedicated versus intrinsic timing somewhat echoes the distinction between absolute, Newtonian time and relational, Leibnizian or Kantian time, respectively: time that is not extracted from its context is by definition not “in and of itself…without reference to anything external,” but rather defined by the process performing the function in which time is intrinsically processed.

It is noteworthy that, while it may appear intuitive for centralized timing mechanisms to be dedicated and for distributed timing mechanisms to be intrinsic, these are orthogonal principles and could theoretically exist in any combination.

Finally, different time scales may be mediated by different mechanisms.^7^ In this review, we will focus on the time scales associated with sensing the passage of time, as measured through tasks requiring judgments of durations or temporal relations among events, in the millisecond-to-second range. Neural timing over this “interval” scale is not well understood, in contrast to circadian rhythms thought to be timed by the suprachiasmatic nucleus using transcriptional feedback loops to generate an approximate 24-h cycle of activity.^11^

### Psychological challenges for a neural clock

There are a few strong motivations for dedicated, centralized timing mechanisms. First, the sense of the passage of time transcends modalities, evidenced by our ability to measure duration across the senses:^9^,^12^,^13^ for example, our ability to compare the duration of a tone to a light, or to reproduce the duration of a visual stimulus with a motor response.^12^ This is suggestive of a common underlying mechanism. Second, individual differences in temporal acuity correlate across perception and action,^14^,^15^ similarly suggesting a common underlying mechanism. Finally, such a dedicated timing mechanism appeals to our intuitions of a consistent, linear passage of time.

However, numerous illusions highlighting distortions in our perception of the passage of time challenge such intuitions. Surprising experimental evidence comes from the study of rapid eye movements (saccades), which we use to foveate objects of interest in the environment. When two bars are flashed sequentially onto a screen around the time that individuals saccade horizontally from one visual target to another, the individuals perceive the duration of separation of the two bars to be significantly shorter than it actually is.^16^ Importantly, perception of auditory clicks presented similarly around saccadic onset suffers no distortion of time. Such evidence is not compatible with a common, unitary sense of linear time, since that would predict that the perception of time across sensory modalities should be unperturbed and equivalent.

In the same vein, it has been demonstrated that, even within the visual modality, temporal perception of different events can be differentially distorted.^17^–^19^ For example, maintaining gaze on a moving stimulus in one portion of the visual field reduces the apparent duration of stimuli subsequently presented to that location, but not to other locations.^17^ Therefore, temporal perception in any given spatial region of the visual field is at least partly independent of temporal perception in other regions––again incompatible with a common, centralized representation of time in the brain.

Additional profound illusions come from studies investigating the perceived duration between one's action and its inferred effect. When individuals are asked to press a button to “cause” a tone, the estimated perceived time between the button press and the tone is reliably shorter than the actual time.^20^ This temporal-compression effect does not occur when the button press and tone occur with identical temporal parameters, but individuals do not believe that their action caused the tone (i.e., when the tones are stated to be randomly generated through a computer program). One prevalent interpretation is that the anticipated, intended consequence of the action is brought closer in time, leading to the labeling of this phenomenon “intentional binding.”^20^ However, some dispute remains over whether perceived intentionality is required or whether the mere perception of causality is sufficient.^21^,^22^

Perceptual distortions between action and inferred effect are also subject to contextual effects of adaptation, which allows the power of these illusions to be revealed. Stetson *et al*.^23^ designed an experiment whereby participants adapted to the delay between pressing a button and perceiving a flash caused by that button press. As before, temporal binding was observed between action and consequence. However, when the adapted delay was suddenly removed, such that the flash occurred almost immediately after the button press, participants perceived the flash as occurring *before* their action that caused it. Such an observation is clearly incompatible with perception being a representation of linearly flowing time, since time becomes inverted to the observer.

Adaptation to temporal delays is further demonstrated in experiments manipulating the timing of information from different sensory modalities. The perception of an external event through more than one sensory channel raises an interesting problem: different sensory channels receive and process information at different speeds, and therefore our perception of these events through different channels should be asynchronous. Indeed, when individuals are asked to judge the order or simultaneity of *separate* brief events perceived through different sensory modalities (for example, a burst of sound and a flash of light), it becomes possible to observe that different sensory channels have different lags to reach perceptual awareness.^24^ However, when we click our fingers or watch someone speaking, we perceive the visual (movement of the fingers, movement of the lips) and auditory input (“snap,” “hello”) to be synchronous. They are correctly perceived to be caused by the *same* external “event,” despite the lags in the different sensory channels. How has the brain solved this problem? It has been suggested that the perceived timing of different sources is dynamically calibrated, such that the multisensory perception of an external event comes to be unitary.

There is empirical evidence for such a calibration process. When participants are exposed to a fixed time lag between auditory and visual stimulation for 3 min, they subsequently show shifts in the time required between auditory and visual stimuli in order to perceive them as simultaneous, known as the “point of subjective simultaneity.”^25^ In other words, participants have adapted to the delay. These adaptation effects also transfer onto other perceptual tasks, indicating that it is a genuine perceptual change. Analogous results have been demonstrated in the context of speech: the point at which participants judge the audio and visual components of audiovisual speech to be simultaneous shifts depending on whether they were previously exposed to asynchronous or synchronous audiovisual components of speech.^26^ Therefore, temporal calibration occurs in the context of simple audiovisual stimuli as well as in more complex and dynamic speech perception in order to generate a unitary perception of stimuli arising from a common external event. Although this does help our perception of external events appear veridical, it requires adaptation processes that distort temporal processing. Again, this is incompatible with a constant mechanism of linear time supporting perception.

### Causation and probability

A common factor that may govern motor–sensory and sensory–sensory temporal distortions is the interpretation of causal relations among events. As alluded to above, the interpretation of causality, and not necessarily intentional action, may give rise to the temporal compression observed between actions and their inferred effects. A string of experiments supports this.^21^,^22^,^27^ For example, by manipulating the belief of causality while controlling for all other variables, Buehner and Humphreys^21^ demonstrated that perceived causality between action and effect is sufficient for temporal compression. Further, when the actor is a mechanical device (an arm of a small machine), instead of self-generated actions, temporal compression still occurs when a causal relation is assumed.^22^ Similarly, the belief that two percepts of different modalities are *caused* by the same external event may be responsible for driving temporal calibration of sensory channels. There is evidence to support this: if participants believe that two sensory percepts originate from a common event, they are more likely to be perceived as simultaneous.^28^–^31^

The role played by “causality” in temporal compression may have its roots in our prior experience with the probable temporal relations between events. Hume proposed that events that are close in space and time are more likely to be perceived as causally related than events further apart.^32^ Drawing insight captured by Bayes’ theorem, events believed to be causally related are more likely to be spatiotemporally close than those that are not. Given some uncertainty in the temporal estimate of an event, the updated temporal estimate will be biased in time toward the other, causally related event.^33^ This Bayesian causal-binding explanation predicts that causal beliefs can lead to temporal compression, but also that temporal compression should be stronger for action–effect pairings that are closer in time, as observed by Haggard *et al*.^20^

The possibility that temporal calibrations are Bayesian in nature has been formally investigated in the context of prior experience. When participants are asked to judge the temporal order of tactile stimuli delivered one to each hand, such that one hand is more likely to receive stimulation before the other, the perceived timing of stimulation follows a shift predicted by a Bayesian integrator.^34^ In other words, learning one hand is more likely to be stimulated before the other biases observers, making them more likely to select stimulation of that hand as occurring before the other in future trials. Analogous mechanisms have been found for audiovisual recalibration.^35^ Importantly, the belief biasing temporal estimates is prior experience of delay, without any necessary mediating role by causal beliefs.

Another important factor influencing calibration is our interaction with the world. When an event is far away, audiovisual asynchrony occurs because of the slower velocity of sound (e.g., fireworks). Parsons *et al*.^28^ demonstrated that active control over a distant audiovisual event renders the auditory and visual percepts more likely to be perceived as simultaneous: we can send out a motor action and analyze the returning multimodal sensations in order to calibrate the relative timing of different modalities. This proposal is rooted in spatial cognition: when individuals wear left–right inverting prism glasses, such that objects on the left appear on the right and vice versa, their vision is highly distorted. However, if allowed to interact with the world by reaching out and touching objects, adaptation occurs and objects on the left appear on the left again.^36^,^37^ Therefore, in both temporal and spatial recalibration, motor interactions with the world, and the feedback received from them, allow dynamic recalibration of our percepts.

It is therefore clear that our perception of time can be tuned dynamically, and can often be highly and locally distorted, at least over a timescale of under a second. This can be observed in the context of foveating events with saccadic eye movements, temporal compression of action consequences, or calibration of different sensory modalities. Therefore, instead of time perception following a constant linear mechanism, it is flexible, dynamic, and continually retuned.

## Neural substrates

Numerous different models of how the brain may keep track of time have been proposed. As noted above, these can be broadly classified as either centralized or distributed, and by the type of timing they address.

### Centralized timing mechanisms

A single, dedicated, centralized, and supramodal clock is the closest model of timing in the brain to keep track of a Newtonian-like “absolute” time. By far the most influential such model is the pacemaker–accumulator model based on an information-processing model of scalar timing theory, consisting of three key components: clock, memory, and decision stages.^38^ A stimulus triggers the accumulator to start counting “ticks,” emitted from the pacemaker based on some sort of periodic neural process, until the end of the timed duration (clock component). The number of counted ticks is then compared in working memory to those previously stored in a reference memory to establish a match (memory component), and thereby make a decision about the duration of the interval (decision component). Timing in this model is therefore achieved by reading out from a timeline generated by the pacemaker.

The pacemaker–accumulator model satisfies the phenomenological motivations discussed above by serving as a centralized, supramodal clock dedicated to timing across all domains. It appeals to our intuitions of a linear passage of time. However, on its own, it is unable to account for the common illusions of temporal perception described above, and support for a direct neural implementation of this model is diminishing.

The pacemaker–accumulator model does have some capacity for flexibility or distortion. For example, it can offer explanation of how states of hyperarousal (e.g., road traffic accidents) are associated with time dilation, since arousal could cause an increased rate of ticks emitted from the pacemaker, accumulating and causing an overestimation of passed time. It can additionally account for how paying attention to the passage of time can influence its apparent rate of passage: attention directed away from the passage of time could cause ticks to be missed by the accumulator, and hence time is underestimated, accounting for how “time flies when you're having fun.” Similarly, focusing on the passage of time could account for how “a watched pot never boils.”^39^–^41^

More problematic for the pacemaker–accumulator model are local distortions in time perception or the temporal alignment between different modalities, including reversals of temporal perception. It allows only for a global speeding or slowing of time perception. It is therefore not possible that a single, centralized timing mechanism is responsible for time perception. Minimally, if a centralized timing mechanism exists, it must interact with other, more localized mechanisms before the perception of timing occurs.^42^,^43^

However, that such a clock cannot account for all mechanisms of time perception in the brain does not rule out a centralized timing system altogether.^43^,^44^ A centralized system could have multiple timing mechanisms running in parallel, allowing modality or spatially specific timing, such as those described in the illusions above. The cerebellum^5^,^6^ and basal ganglia^7^ have been proposed as candidate brain structures for timing in the millisecond to the hundreds-of-milliseconds ranges, respectively. These brain areas have structural architectures compatible with multiple, parallel timing mechanisms: the regular repeating architecture of the cerebellum and the repeating loops involving the basal ganglia and cortical structures (corticostriatal loops). In a centralized system with plural mechanisms, the timing of any given event could be independent of another: for example, timing of a visual stimulus in a given spatial location compared to one in another.

### The cerebellum as a centralized timer

There is evidence indicating that the cerebellum may play a key role in timing within the millisecond to hundreds-of-milliseconds ranges.^5^ A recent meta-analysis of multiple neuroimaging studies^45^ revealed that the cerebellum, among other areas, is active for both perceptual and motor timing tasks of subsecond duration. This observation is supported by deficits when cerebellar function is impaired. Both patients with lesions in the cerebellum and normal subjects who receive transient magnetic pulses to disrupt ongoing neural activity in the cerebellum exhibit impaired timing behaviors in both motor^46^–^48^ and perceptual^47^,^49^,^50^ subsecond timing tasks. The consistent activation and necessity of the cerebellum in the timing of subsecond durations across a range of tasks has led to the suggestion that it performs common computations dedicated to temporal processing of these durations.^6^,^8^

An example of the specific neural mechanisms that may underlie timing in the cerebellum proposes that time is encoded in a large population of active neurons.^51^ A stimulus causes the activity of a large population of extremely abundant neurons, known as granule cells, to change dynamically, owing to negative feedback loops between them and interneurons. In this way, time is encoded in the activity of these neurons, and can be read out by neurons on which granule cells converge, known as Purkinje cells. Purkinje cells will fire when they recognize certain patterns of activity across the neurons converging upon them, and the pattern of activity they recognize is determined by the weights between the Purkinje cell and granule cell synapses.^51^,^52^ In this way, time is decoded from the time-varying activity of a large population of neurons.

However, despite being a localized brain area, apparently performing common computations for temporal processing across different tasks, the cerebellum is unlikely to be a dedicated clock. The cerebellum has also been strongly implicated in motor control and associative learning.^53^ Dissociating whether timing or other functions are the primary computational purpose of the cerebellum is difficult. Indeed, there is debate in the cerebellum literature as to whether the cerebellar cortex is responsible for storing memory traces of temporal^54^ or conditioned–unconditioned stimulus^55^ relationships.

### The basal ganglia as a centralized timer

Converging evidence suggests that the subcortical structures known as the basal ganglia may play an important role in timing. For example, damage to the striatum—a part of the basal ganglia—in Parkinson's disease is associated with robust and replicable deficits in temporal tasks.^56^ Dopamine is one of the key neurotransmitters stimulating the striatum, and its dysregulation similarly causes temporal distortion in this disease, as well as in schizophrenia.^56^ Corresponding pharmacological and lesion studies in animals further implicate the striatum and dopamine system in timing.^7^ Functional neuroimaging data in humans reveal activity in the basal ganglia and connected cortical areas during timing tasks.^57^,^58^ For example, when participants pay attention to the duration versus color of a stimulus, there is an increase in activity in a corticostriatal network.^59^ Based on these converging findings, a model of how the basal ganglia and associated neural circuitry may be able to tell time has been proposed.

The *striatal beat frequency* (SBF) model proposes that duration estimation is based on the detection of coincidental oscillatory processes in corticostriatal circuits.^7^ The model suggests that, at the beginning of an event to be timed, dopaminergic output from the ventral tegmental area “resets” and synchronizes corticostriatal oscillatory activity. Neurons in the striatum, onto which cortical neurons converge, then track the oscillatory activity until a reinforcement signal indicates the end of the timed duration. This reinforcement signal, originating from the substantia nigra, releases dopamine into the striatum, which modulates corticostriatal synaptic weights. With experience, the striatal neurons learn to recognize a specific “snapshot” of coincidental oscillatory activity, representing a specific duration. This model predicts, for example, that a peak in neuronal activity in the striatum should be observed around the end of a timed duration—a finding paralleled by ensemble recordings of striatal neurons.^60^

Some have proposed the SBF model to be a dedicated timer.^57^ However, it could be argued that frontostriatal systems are far from exclusively dedicated to timing functions, as they may instead/also be important in action selection and in sensorimotor decision making.^6^

Importantly, the SBF model and the cerebellar timing model outlined above do not require any temporal accumulator. Instead, they rely on coincidence detection—a neurophysiologically more feasible computation.^44^ The models bear striking similarities. In both, massive convergence onto a set of neurons (Purkinje cells in the cerebellum, striatal neurons in the basal ganglia) allows these neurons to recognize snapshots of neural activity that evolves over time. Further, in both, reinforcement signals (transmitted by climbing fibers in the cerebellum and dopaminergic nigrostriatal neurons in the basal ganglia) teach Purkinje (cerebellum) and striatal (basal ganglia) neurons to recognize “stamps” of large-scale, time-dependent neural activity by adjusting synaptic weights.

To summarize, it is possible that the basal ganglia and cerebellum serve as centralized clocks, despite the evidence of localized timing mechanisms. However, it is not clear whether these timing mechanisms are dedicated to the problem of timing, or whether timing is a process inextricably wrapped up in other computational analyses being performed: for example, associative learning in the cerebellum or selection in the basal ganglia.

### Distributed, intrinsic timing mechanisms

Some scholars have disposed of centralized timing mechanisms altogether in favor of distributed timing mechanisms. In these models, temporal processing is an intrinsic property of information processing and distributed throughout the brain. One may ask, why dispose of a centralized clock? Perhaps, simply because we can: centralized clocks may not be necessary for coding temporal information. Additionally, neuronal activity that covaries with the timing of events has been observed in diverse areas of the brain, suggesting that it may be a ubiquitous aspect of cortical processing. In the proposed distributed timing mechanisms outlined below, time is intrinsically represented: neural networks inherently encode time while processing other information, such as stimulus features or motor commands.

#### State-dependent networks (SDN)

If there is no centralized clock, how do we process the temporal dimension of events? An intriguing possibility is that a neural network processing a sensory event inherently encodes its temporal component as a result of time-dependent changes in network state.^10^,^61^

This SDN model proposes that local neural circuits are inherently capable of processing both temporal and spatial information and that the processing of both is inextricably linked.^61^ As a result, temporal information is inherently encoded in evolving neural trajectories—analogous to the spatiotemporal pattern of ripples in a pond^61^ (one could determine how long ago the stone was dropped by looking at the trajectory of the ripples). It is noteworthy that the SDN model likely accounts for temporal processing only up to durations of a few hundred miliiseconds.^10^

A crucial aspect of the SDN model is that spatiotemporal information is encoded in local neural circuits,^10^ and timing is therefore distributed throughout the brain. This type of mechanism would be compatible with the findings discussed above demonstrating spatially localized temporal distortions,^17^–^19^ since if time is encoded in local neural networks, then distortions of time in one location can be independent of those in another. The SDN model therefore suggests that temporal processing over short time scales occurs in local sensory or motor cortices, consistent with functional neuroimaging data.^62^

#### Climbing neural activity

Another influential distributed account for how the brain may keep track of time is through climbing neural activity.^63^ This model suggests that neural activity increases as a function of time, ramping up until the end of a timed duration. The existence of climbing neural activity has been documented through recordings from individual neurons in numerous brain areas of monkeys. These include the anterior cingulate cortex, premotor cortex, posterior parietal cortex, supplementary motor area (SMA), and pre-SMA (for review, see Ref. 63). Such activity is generally elicited by a stimulus signaling the onset of a fixed temporal interval preceding a response or another task-relevant stimulus.

The ramping up of neural activity until the end of a timed duration is suggestive of such neurons generating an internal representation of duration.^63^,^64^ Interestingly, while this may be true, it cannot be determined that such activity is performing an exclusively temporal function, representing duration per se. Since the end of the timed duration is invariably marked by a response or another stimulus, the ramping up of neural activity could represent signals related to motor preparation or expectancy, and not necessarily timing per se.

For example, it has been shown that neurons in a region of the parietal cortex of the monkey, which has been implicated in attention and decision making, increase their firing rate as a function of elapsed time in anticipation of a salient event. In this case, the neuron's firing rate, as well as representing elapsed time, also represents the probability of the event occurring given that it has not already occurred,^65^ also known as the hazard function. The neuron, therefore, may represent mounting expectation instead of or in addition to timing of the interval. Similarly, in another experiment, the apparent coding of time by neurons in this parietal region may represent decision variables. Monkeys were trained to judge the duration of a stimulus as longer or shorter than another by making a saccade to targets representing “shorter” or “longer.”^66^ Neurons with a receptive field containing the “shorter” target maintained their activity until the duration of the stimulus exceeded that of the stimulus to be judged against, after which activity dropped off. Activity in neurons with the “longer” target in their receptive field increased over time. Therefore, although representing time, these neurons also represent the monkey's choices. These two examples demonstrate how apparent coding of duration can be inextricably tied to other variables.

Human studies have similarly suggested that neural activity apparently representing duration may reflect other processes. For example, a slowly developing negative potential recorded from the scalp of participants using electroencephalography—the contingent negative variation—is present during the timing of intervals.^67^ It develops when a response is required at a predictable point in time, thereby requiring temporal estimation. Accordingly, some have interpreted it to reflect a neural representation of the accumulator in the pacemaker–accumulator model.^68^ However, critical evaluation of the evidence on which this proposition is based suggests that the contingent negative variation does not index interval timing mechanisms, but rather expectation, response preparation, or decision-making processes that are time dependent.^63^,^69^,^70^

Further, Cui *et al*.^71^ asked participants to react as quickly as possible to a cue following a variable waiting period, and investigated neural correlates of this waiting period using functional magnetic resonance imaging (fMRI). It was found that the amplitude of the fMRI signal in the SMA after the cue presentation depended on the wait time, resembling the cumulative hazard rate (i.e., the cumulative probability of cue presentation given it has not already been presented). Importantly, when participants were informed of the cue arrival time, the fMRI signal no longer depended on the wait time, suggesting that the signal does not simply reflect elapsed time, but rather temporal probabilities. The authors suggested that this signal may be produced by the integration of hazard signals from the parietal cortex.^65^ Therefore, the apparent representation of duration by climbing neural activity is likely inextricably and intrinsically linked to other variables.

One may wonder whether, in order for neurons to ramp their activity as a linear function of time, they require input from a centralized system. While this is possible, an alternative explanation is that such activity could be intrinsically generated,^9^ thereby negating necessity for a centralized system at any stage of processing. Indeed, it has been proposed that self-sustaining, time-varying neural activity can be generated by the internal dynamics of recurrently connected networks.^9^ A model based on these principles is, of course, entirely intrinsic in nature, and has recently been applied to account for motor timing.^9^

### Models of simultaneity and recalibration

In addition to interval timing, neural models have been proposed to account for the temporal recalibration effects. Cai *et al*.^72^ have developed a model for recalibration of temporal order between motor and sensory events. In their model, different neuronal populations encode different motor–sensory delays, and the output of these neurons reaches opponent neuronal populations encoding the sensory event as “before” or “after” the motor event. The difference in activity between these “before” and “after” populations determines the perceptual judgment. The input weights to the “before”/“after” neuronal populations are scaled by adaptation to temporal delays. For example, if repeatedly exposed to a delay between motor actions and their consequences, the input weights of the “after” population will decrease, while those of the “before” population will increase. Therefore, the system becomes biased toward making “before” decisions. This would lead to a shortening of the perceived time between action and effect, as observed in the motor–sensory recalibration experiments described above. If the delay is then suddenly removed, the participant will still be biased to making a “before” decision, leading to the illusory reversal of action and sensation, as is observed experimentally.^23^ An analogous model has been proposed for sensory–sensory audiovisual recalibration.^73^

It is noteworthy that, in these models, temporal recalibrations do not result from changes in signal transmission or processing times, which have been proposed as alternative mechanisms of recalibration.^74^–^76^ Rather, the temporal properties of events themselves are represented. In this way, temporal perception is the brain's interpretation of external events, and not simply the order in which events occur in time, as our intuition would suggest (which is why we find the illusions so striking). For example, audiovisual recalibration is attributable to adjusting the weights of neurons tuned to different delays onto output populations, as opposed to altering the signal transmission or processing time of auditory or visual signals. This moves away from the time-encodes-time notion, whereby the “external” timing of events encodes the perceptual timing by the relative signal arrival times. Consistent with the hypothesis that temporal perception is not a simple reflection of simultaneity of neural signals, but rather the brain's interpretation of external events, Nishida and Johnston^77^ have proposed “time markers,” which refers to the representation of temporal patterns; it is the relationship between these representations that gives rise to the perception of the relative timing of events.

These ideas echo those of Dennett and Kinsbourne,^78^ who draw the distinction between the representation of temporal properties and the temporal properties of representations. According to their scheme, for the brain to represent “A before B,” a representing of A before a representing of B is not required,^78^ as is observed in the recalibration experiments. The authors point out that this accounts for the well-known “rabbit illusion”—whereby a rapid sequence of taps delivered near the wrist and then the elbow creates the illusory sensation of sequential taps hopping up the arm toward the elbow, before the elbow stimulation is perceived. That time does not necessarily encode time allows the “backdating” in time required for the perception of taps between the wrist and elbow before the perception of taps at the elbow.

However, there appears to be an upper bound on the durations susceptible to temporal compression or calibration.^20^,^23^ This may reflect a maximum separation before two events are no longer labeled as causally related. Alternatively, it could reflect a temporal window in which the temporal realignments could still be useful for the brain to control action.^78^ Once the perceptual processes within the observer have begun to perform their function (e.g., initiating action), there is no point in altering the representations.

Beyond the “interval” time scale, the story may become very different. Longer durations—above the hundreds-of-milliseconds range probed in the above experiments—do appear to follow the arrow of time more linearly: you know you read this paragraph after the previous, and, so far, we have discovered no illusion that could shift this.

### Other considerations

#### Is timing ever truly dedicated?

A deep issue regarding timing in the brain is whether neural processing can ever be said to be dedicated to the problem of timing, or whether temporal processing is always, by necessity, wrapped up with the coding of other attributes in the brain. As discussed above, even in apparently dedicated models such as in cerebellar timing and the SBF model, timing may actually be a by-product of coding other processes, such as associating stimuli to actions or selecting motor commands.

Other models, such as those directly coding temporal delay to determine the order of events, may appear to be dedicated. However, it is interesting to speculate whether such models really necessitate a pure representation of time. Could time-related activity in these neurons coding “before”/“after” also be inextricable from the coding of other, task-relevant properties? For example, we have indicated that climbing neural activity could also reflect decision variables rather than interval timings. Analogously, neural activity in these models could encode the decision variable “before” or “after” as part of the choice-selection process. In other words, neural activity linked to timing could reflect a process of evidence accumulation for action selection, and not the extracted representation of time per se.

The question remains whether the coding of temporal processing in the brain can ever truly be separated from its context. What purpose could it have, since perhaps only timing in the context of something else could bear on our perception and action toward external events, as well as on the memories and musings of our internal mental life? Indeed, it is difficult to conceive of a completely abstracted and disembodied neural representation of time, although this on its own may be insufficient ground for denying its possible existence.

#### Time and space

At multiple levels of analysis, parallels can be drawn between temporal and spatial processing. For example, in addition to the saccade-induced temporal distortions discussed above, saccades also distort spatial perception,^79^ with dynamics that are tightly coupled to the temporal distortions.^80^ Accordingly, models such as the SDN model explicitly acknowledge and propose the inextricable bundling of temporal and spatial information.^61^

The model by Cai *et al*.^72^ using delay lines to account for temporal recalibration also proposes the overlap of temporal and spatial processing. Recalibration of temporal order judgments is proposed to be a temporal analogue to the motion after effect, with identical neural mechanisms underlying judgments of both time and space.^72^ In other words, the neural computations performed are the same, suggesting an evolutionarily conservative genetic program coding for a module capable of dealing with either time or space depending only on the inputs.^72^ Further, as discussed above, there are apparent similarities in the way the brain uses motor interactions with the world and the feedback received from them to calibrate temporal and spatial percepts. Finally, parallels are increasingly proposed between “place” and “time” cells identified in the rodent hippocampus, which are thought to contribute to the spatial and temporal integration and organization of events in memory.^81^–^83^

An interesting question that can be raised about space, given the debates about timing, is whether neural mechanisms are ever dedicated to spatial processing. Or do similar arguments against dedicated timing hold for space? For example, place cells, which code the location of an animal in space,^84^ may not code the abstract representation of space per se, but rather the structured relation among things in space. This is supported by the fact that spatial coding in place cells is highly context dependent: cells code different locations in different environments, and their mapping changes when the environment changes.^85^ Furthermore, spatial properties of neurons in parietal and premotor regions within sensorimotor circuits are often linked to locations relative to motor effectors, as opposed to absolute space.^86^,^87^

Taken together, it is interesting to speculate whether there is a commonality in the way that the brain deals with temporal and spatial information to create a meaningful and coherent perception of the world around us. The similarities in the way that the brain deals with these dimensions raise the possibility that the same neural architectures could process analogous aspects of both. Furthermore, if time and space are not coded in isolation in the brain, but instead can only be explained as the relation between things, could this contribute to philosophical debates about the reality and nature of these dimensions? Kant, as mentioned above, questions whether both time and space are constructs of the mind, neither of which may have a real external existence—a stance somewhat echoed by Einstein, who states: “Time and space are modes by which we think and not conditions in which we live.” One suggestion is that they are both constructs that help organize our perception of events, so that we can better understand our world and the relations of events within it.

## Conclusion

Experimental psychology and neuroscience have, like physics, challenged some of our intuitive notions of a constant and linear passage of time, and have begun to elucidate some of the mechanisms by which the brain may represent time. At least over a short time frame, our temporal perception of events is far from veridical, and multiple timelines are capable of dynamic recalibration. This is incompatible with the notion of a unitary centralized and dedicated clock, from which all timing is performed. Alternative accounts for multiple centralized clocks and distributed timing mechanisms have been proposed. These models are not mutually exclusive, and timing may also be achieved through a combination of centralized and distributed processing.^42^,^43^ Regardless of the exact timing structures and mechanisms, the fundamental question remains whether neural processing is ever exclusively dedicated to the problem of timing. Neural activity that may appear as such can always be reframed to be coding some other process that occurs *in* time, rather than time itself.
